# Platinum-Based Neoadjuvant Chemotherapy for Breast Cancer With BRCA Mutations: A Meta-Analysis

**DOI:** 10.3389/fonc.2020.592998

**Published:** 2020-11-09

**Authors:** Chang-Jun Wang, Ying Xu, Yan Lin, Han-Jiang Zhu, Yi-Dong Zhou, Feng Mao, Xiao-Hui Zhang, Xin Huang, Ying Zhong, Qiang Sun, Cheng-Gang Li

**Affiliations:** ^1^ Department of Breast Surgery, Peking Union Medical College Hospital, Beijing, China; ^2^ Department of Dermatology, University of California, San Francisco, CA, United States; ^3^ State Key Laboratory of Medicinal Chemical Biology, Nankai University, Tianjin, China; ^4^ College of Pharmacy, Nankai University, Tianjin, China

**Keywords:** breast cancer, platinum, BRCA gene, neoadjuvant chemotherapy, pathological complete response

## Abstract

**Background:**

Triple-negative breast cancer (TNBC) is one of the most aggressive subtypes of breast cancer and the major phenotype of BRCA related hereditary breast cancer. Platinum is a promising chemotherapeutic agent for TNBC. However, its efficacy for breast cancer with BRCA germline mutation remains inconclusive. Here we present a meta-analysis to evaluate the effect of platinum agents for breast cancer patients with BRCA mutation in neoadjuvant setting.

**Materials and Methods:**

Pubmed, Embase, and Cochrane Central Register of Controlled Trials databases were searched for relevant studies on neoadjuvant platinum treatment and BRCA related breast cancer. Fixed- and random-effect models were adopted for meta-analyses. Heterogeneity investigation was conducted by sensitivity and subgroup analyses. Publication bias was evaluated by funnel plot and Begg’s test.

**Results:**

In all, five studies with 363 patients were included for meta-analysis. The pooled pathological complete response (pCR) rates were 43.4% (59/136) and 33.9% (77/227) for platinum and control groups, respectively. Adding platinum to neoadjuvant regimen did not significantly improved pCR rate (odds ratio [OR]: 1.340, 95% confidence interval [CI] = 0.677–2.653, *p* = 0.400). Sensitivity analyses also revealed platinum did not significantly increase pCR rate in either TNBC or HER2- patients (TNBC subgroup: OR: 1.028, 95% CI = 0.779–1.356, *p* = 0.846; HER2- subgroup: OR: 0.935, 95% CI = 0.716–1.221, *p* = 0.622).

**Conclusions:**

Our meta-analysis suggested that the addition of platinum to neoadjuvant chemotherapy did not significantly improve pCR rate for patients with BRCA mutations. Further large-scale randomized control trial with survival data may provide more robust evidence on therapeutic value of platinum for breast cancer neoadjuvant treatment.

## Introduction

Triple-negative breast cancer (TNBC) comprises up to 17% of all breast cancer and remains the major phenotype of hereditary breast cancer (1). The most prevalent causes for hereditary breast cancer are germline pathogenic alterations in two major breast cancer susceptibility genes (BRCA1 [OMIM 113705] and BRCA2 [OMIM 600185). As the clinical application of gene sequencing, the proportion of pathogenic or likely pathogenic germline BRCA1/2 variants carriers increases accordingly. And around 70% of BRCA1 and 23% of BRCA2 related breast cancer were TNBC (2).

BRCA1 and BRCA2 are two critical tumor suppression genes for repair double-stranded DNA breaks by homologous recombination (3). Heterozygous germline inactivation followed by disfunction of the other normal allele as a “second hit” would lead to homologous recombination deficiency and vulnerability of cancer cells to DNA damage (4). Thus, the application of DNA-damaging agents, such as platinum, in BRCA related cancer arouse great interest. The mechanism of platinum agents lies in its covalent binding with DNA which leads to DNA damage. As the DNA damage accumulates and the damage burden exceeds the limit of DNA repair, cancer cell could not maintain normal mitosis and ultimately undergo apoptosis (5, 6).

Several trials demonstrated TNBC could benefit from platinum-containing neoadjuvant treatment. The GeparSixto trial assessed the efficacy of adding carboplatin to anthracycline and taxane based regimen. In TNBC subgroup, the pathological complete response (pCR) rate of carboplatin group reached 53% and significantly higher than control group (37%) (7). Although GeparSixto trial was criticized for adopting a non-standard regimen, its results were further validated in the CALGB 40603 trial. CALGB 40603 trial enrolled 443 TNBC patients and adopted a standard neoadjuvant regimen with weekly paclitaxel followed by dose-dense doxorubicin plus cyclophosphamide with the addition of carboplatin and/or bevacizumab. Carboplatin group reached pCR rate up to 54% compared to control group with 41%. Both trials shed light on the clinical application of platinum for TNBC (8).

However, the efficacy of neoadjuvant platinum for patients with BRCA mutation remains inconclusive. Study by Byriski et al. reported extremely high pCR rate (83.3%) with single agent cisplatin (9). On the contrary, GeparSixto trial and recently published INFORM trial demonstrated the pCR rate of BRCA mutation carriers was not further improved with the application of platinum (10, 11). Therefore, we conducted this meta-analysis to evaluate the therapeutic effect of platinum in neoadjuvant therapy for breast cancer with BRCA mutations. This is the first meta-analysis to investigate the impact of platinum within BRCA mutated population.

## Materials and Methods

### Literature Search

The following databases were searched for relevant studies: PubMed (from 1946 to June, 2020), Embase (host: Ovid, from 1947 to June, 2020) and Cochrane Central Register of Controlled Trials (CENTRAL, from 2000 to June, 2020). The following medical subject headings and keywords were used: “platinum,” “carboplatin,” “cisplatin,” “neoadjuvant,” “pre-operation,” “breast cancer,” “breast neoplasm” or “breast carcinoma,” and “BRCA”. No limitation was set regarding languages or regions of publications. All the relevant references were retrieved and manually screened to ensure the sensitivity of the literature search.

### Data Extraction

A predesigned data extraction form was used by two reviewers (C-JW, YL, and Y.X) for collecting data. The following characteristics of included studies were extracted for subgroup and sensitivity analyses: authors; publication year; country; number of patients; stage; breast cancer subtype; study design; study population; BRCA mutation status; treatment; platinum used; pCR definition; pCR rate; number of patients who achieved pCR in both experimental and control groups were extracted from tables or text of included studies.

### Selection Criteria and Quality Assessment

The following inclusion criteria were set to select eligible studies: studies that included patients with BRCA1/2 mutations or provided data on BRCA1/2 mutation patients as a subgroup; assessment of BRCA1/2 mutation as germline mutation; early or locally advanced breast cancer patients receiving neoadjuvant systemic treatment; available data of response indicators for neoadjuvant chemotherapy (pCR, residual cancer burden [RCB] and so on). Exclusion criteria were set as follow: less than 10 patients with BRCA mutations were included; metastatic breast cancer; review, meta-analysis, editorial, letter, case reports, guidelines and study protocols.

The titles and abstracts of all the citations were manually evaluated as initial screening. The study eligibility was independently assessed by two reviewers (C-JW and YX) according to the inclusion/exclusion criteria. Then, full texts of the potentially relevant studies were retrieved and reviewed for inclusion by the same two reviewers. Disagreement was resolved by consensus (C-JW, YX, YL, QS, and C-GL).

STROBE checklist was adopted for quality assessment of the included studies (12). Ordinal scale from 1 to 5 (1 = Worst, 5 = Best) was used to score each item in the STROBE Checklist by two independent reviewers (C-JW and YL). The final quality scores (QS) were the mean of scores generated by each reviewer with higher values indicating a better methodological quality. The mean of the QS of all the included studies was set as the cutoff to differentiate low- and high-quality subgroups.

### Statistical Analysis

The demographic and clinicopathological parameters were presented as means and proportions, and between group differences were assessed by Pearson Chi-square test. The odds ratio (OR) of pCR was set as the primary analytical endpoint. Statistical variables such as OR and pCR percentage were directly taken from the full-text articles and used for meta-analysis. Fixed or random effects models were used based on whether significant heterogeneity existed between included studies.

Heterogeneity was assessed by Cochrane’s Q and I-square statistics. Cochrane’s Q test with *p* < 0.05 or I-square > 50% was considered to have significant heterogeneity and random effect model was used for meta-analysis. Otherwise, fixed effect model would be appropriate. Subgroup and sensitivity analyses were performed for heterogeneity investigation. Publication bias was assessed by funnel plot symmetry and Begg’s test.

All the statistical tests were two-sided, and statistical significance was defined as *p* < 0.05. Statistical analyses were conducted by STATA version 16.0 (Stata Corporation, College Station, TX, USA).

## Results

**Figure 1:**
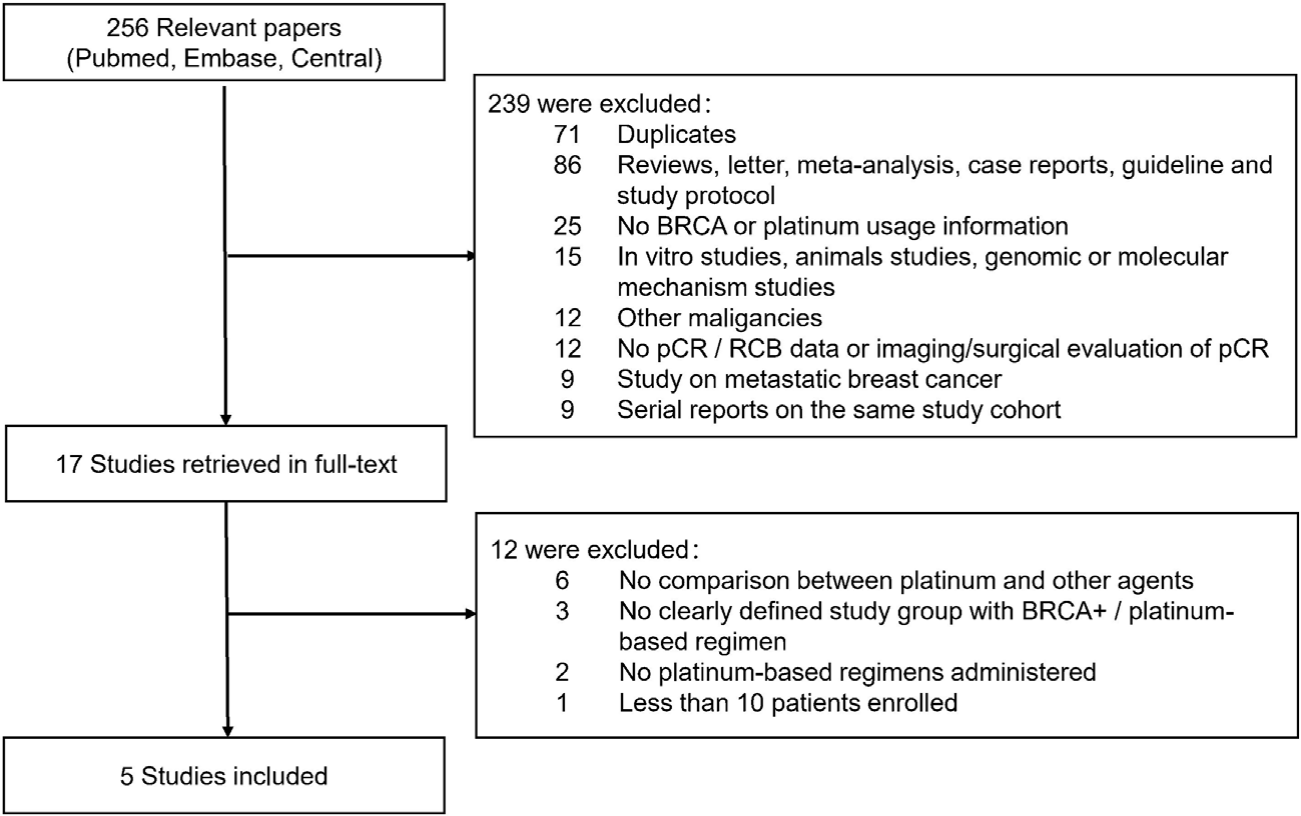
Flowchart of articles reviewed and included in meta-analysis. pCR, pathological complete response; RCB, Residual cancer burden.

In all, 256 relevant citations were found in Pubmed, Embase and CENTRAL Database for initial screening, and 239 citations were excluded for not fulfilling the inclusion/exclusion criteria. Seventeen citations were considered to be potentially relevant to the study objective and full-text articles were retrieved for further evaluation. Finally, five studies with 363 patients were included for meta-analysis (9–11, 13, 14). The flowchart for literature search and selection was showed in **Figure 1**. **Supplementary Table 1** showed the QS of included studies.

### Characteristics of Included Studies and Study Population

**Table 1** summarized the main characteristics of included studies. All the studies included early breast cancer patients, two studies included Stage I-III patients (9, 14), and the other three included Stage II-III patients (10, 11, 13). Three studies included only TNBC (10, 13, 14) and one study included HER2- patients (11). For study design, study by Hahnen et al., Loible et al., and Tung et al. were randomized controlled trial (RCT) (10, 11, 13), and the other two were prospective cohort studies (PCS) (9, 14). Two studies involved merely BRCA1 mutated patients (9, 14), while the other three involved both BRCA1 or 2 mutated patients (10, 11, 13). For platinum usage, two studies used cisplatin as single agent regimen (9, 11), and the other three used carboplatin in combination with the other agents (10, 11, 13). All the included studies adopted “ypT0/isN0” as the definition of pCR.

**Table 1:** Characteristics of studies included in meta-analysis.

Study	N	Stage	Subtype	Study design	QS	Study population	BRCA status	Treatments	Platinum	pCR definition	pCR rate	
											Platinum	Control
Byrski et al. (9)	102	I-III	NS	PCS	Low	Whole cohort	BRCA1	**TG:** Cisplatin 75mg/m^2^ **CG:** CMF/AC/FAC/AT	Cisplatin	ypT_0/is_N0	83.3% (10/12)	15.6% (14/90)
Hahnen et al. (13) **(GeparSixto)**	50	II-III	TNBC	RCT Phase II	High	Subgroup	BRCA1 & 2	**TG:** P + Dox + Bev + Cb **CG:** P + Dox + Bev	Carboplatin	ypT_0/is_N0	65.4% (17/26)	66.7% (16/24)
Loible et al. (10) **(BrighTNess)**	46	II-III	TNBC	RCT Phase III	High	Subgroup	BRCA 1 & 2	**TG:** P + Cb-AC **CG:** P-AC	Carboplatin	ypT_0/is_N0	50.0% (12/24)	40.9% (9/22)
Sella et al. (14)	48	I-III	TNBC	PCS	Low	Subgroup	BRCA1	**TG:** ddAC-wT + Cb **CG:** ddAC-T	Carboplatin	ypT_0/is_N0	64.3% (9/14)	67.6% (23/34)
Tung et al. (11) **(INFORM)**	117	I-III	HER2-	RCT Phase II	High	Whole cohort	BRCA1 69% BRCA2 30% Both 2%	**TG:** Cisplatin **CG:** AC	Cisplatin	ypT_0/is_N0	18.3% (11/60)	26.3% (15/57)

AC, Doxorubicin 60mg/m^2^ and cyclophosphamide 600mg/m^2^; AT, Doxorubicin and docetaxel; CG, Control group; CMF, Cyclophosphamide, methotrexate, and fluorouracil; ddAC-wT + Cb, dose dense doxorubicin 60 mg/m^2^ and cyclophosphamide 600 mg/m^2^ every 2 weeks with G-CSF support followed by 12 weekly cycles of paclitaxel 80/m^2^ with carboplatin AUC 1.5;

ddAC-T, dose dense doxorubicin 60 mg/m^2^ and cyclophosphamide 600 mg/m^2^ every 2 weeks with G-CSF support followed by dose dense paclitaxel 175/m^2^ 2 weekly for four cycles or 12 weekly cycles of paclitaxel 80/m^2^; FAC, Fluorouracil, doxorubicin, and cyclophosphamide; NS, Not specified; pCR, pathological complete response; PCS, Prospective cohort study; P + Cb - AC, Paclitaxel 80 mg/m^2^ weekly plus carboplatin AUC 6 every 3 weeks for 12 weeks followed by doxorubicin 60 mg/m^2^ plus cyclophosphamide 600 mg/m^2^ every 2 or 3 weeks; P – AC, Paclitaxel 80 mg/m^2^ weekly followed by doxorubicin 60 mg/m^2^ plus cyclophosphamide 600 mg/m^2^ every 2 or 3 weeks; P + Dox + Bev + Cb, Paclitaxel 80 mg/m^2^ plus nonpegylated liposomal doxorubicin 20 mg/m^2^, both once a week for 18 weeks plus bevacizumab 15 mg/kg intravenously every 3 weeks simultaneously plus carboplatin at a dose of 2.0 AUC, once every week for 18 weeks; P + Dox + Bev, paclitaxel 80 mg/m^2^ plus nonpegylated liposomal doxorubicin 20 mg/m^2^, both once a week for 18 weeks plus bevacizumab 15 mg/kg intravenously every 3 weeks simultaneously with all cycles; QS, Quality score; RCT, Randomized control trial; TG, Test group; TNBC, Triple negative breast cancer.

The demographic and clinicopathological parameters of included study population were listed in **Table 2**. The T stage (*p* = 0.265), N stage (*p* = 0.201) and estrogen receptor status (*p* = 0.139) were comparable between platinum and control groups. The platinum group involved more ductal cancer than control group (80.5 vs. 66.5%, *p* = 0.043).

**Table 2:** Demographic and clinicopathological characteristics of study population.

		Platinum group(N = 72)	Control group(N = 148)	*P* value
**Age^#^**		40.6	42.7	
**T stage**				0.265
	**T1**	16 (22.5%)	21 (14.0%)	
	**T2**	39 (55.0%)	95 (63.3%)	
	**T3**	16 (22.5%)	34 (22.7%)	
**N stage**				0.201
	**N0**	37 (51.4%)	61 (41.2%)	
	**N1-3**	35 (48.6%)	87 (58.8%)	
**ER status**				0.139
	**Positive**	20 (32.8%)	35 (22.0%)	
	**Negative**	41 (67.2%)	124 (78.0%)	
**Histology**				0.043
	**Ductal**	58 (80.6%)	105 (66.5%)	
	**Others**	14 (19.4%)	53 (33.5%)	

ER, Estrogen receptor.

^#^The standard deviation of patient age was unable to calculate due to insufficient data provided by included studies. Thus, statistical analyses could not be performed.

### The Impact of Platinum on pCR Rate for Patients With BRCA Mutation

**Figure 2:**
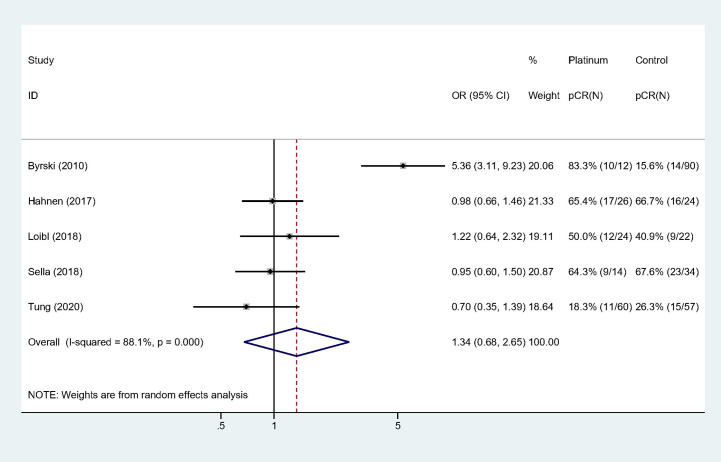
Forest plot of OR for pCR rate.

All the studies reported pCR rate and the pooled pCR rate were 43.4% (59/136) and 33.9% (77/227) for platinum and control groups, respectively. There was significant heterogeneity among the included studies (Cochrane’s Q *p* < 0.001, I-square = 88.1%). Adding platinum to neoadjuvant regimen did not significantly improved pCR rate (OR: 1.340, 95% confidence interval [CI] = 0.677**–**2.653, *p* = 0.400) (**Table 3**). **Figure 2** showed Forest plot of OR for pCR rate and heterogeneity among included studies.

**Table 3:** Overall results and subgroup/sensitivity analyses.

Variables		Heterogeneity		Meta-analyses		
		I-square	*p* value	Model	OR (95% CI)	*p* value
**Overall**		88.1%	<0.001	Random	1.340 (0.677**–**2.653)	0.400
**Stage**	I - III	93.6%	<0.001	Random	1.535 (0.433**–**5.441)	0.507
	II - III	0.0%	0.557	Fixed	1.068 (0.754**–**1.512)	0.711
**Subtype**	HER2-	0.0%	0.698	Fixed	0.935 (0.716**–**1.221)	0.622
	TNBC	0.0%	0.800	Fixed	1.028 (0.779**–**1.356)	0.846
Study type^*^	PCS	95.7%	<0.001	Random	2.241 (0.410**–**12.259)	0.352
	RCT	0.0%	0.487	Fixed	0.930 (0.674**–**1.283)	0.659
Quality Score^*^	Low	95.7%	<0.001	Random	2.241 (0.410**–**12.259)	0.352
	High	0.0%	0.487	Fixed	0.930 (0.674**–**1.283)	0.659
**Study population^&^**	Subgroup	0.0%	0.800	Fixed	1.028 (0.779**–**1.356)	0.846
	Whole cohort	96.1%	<0.001	Random	1.950 (0.212**–**17.913)	0.555
BRCA status^*^	BRCA1 only	95.7%	<0.001	Random	2.241 (0.410**–**12.259)	0.352
	BRCA1 & 2	0.0%	0.487	Fixed	0.930 (0.674**–**1.283)	0.659
**Experiment regimen^&^**	Combination	0.0%	0.800	Fixed	1.028 (0.779**–**1.356)	0.846
	Single	96.1%	<0.001	Random	1.950 (0.212**–**17.913)	0.555
**Platinum^&^**	Carboplatin	0.0%	0.800	Fixed	1.028 (0.779**–**1.356)	0.846
	Cisplatin	96.1%	<0.001	Random	1.950 (0.212**–**17.913)	0.555

CI, Confidence interval; OR, Odds ratio; PCS, Prospective cohort study; RCT, Randomized control trial; TNBC, Triple negative breast cancer.

*Collinearity existed among these three variables, so the subgroup analyses based on these three variables drew the same conclusion.

^&^Collinearity existed among these three variables, so the subgroup analyses based on these three variables drew the same conclusion.

#### Subgroup and Sensitivity Analyses

The results of subgroup and sensitivity analyses were listed in **Table 3**.

**Figure 3:**
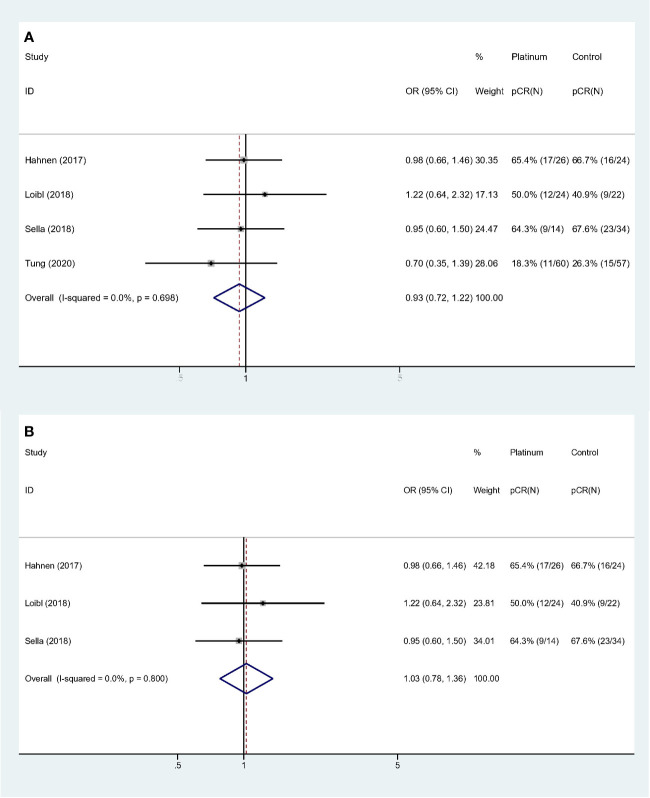
Sensitivity analyses of HER2- and TNBC patients: **(A)** HER2-; **(B)** TNBC.

For stages of disease, studies included either Stage I–III or II**–**III did not reach statistically significance to confirm the improvement of pCR rate (Stage I**–**III: OR: 1.535, 95% CI = 0.433**–**5.441, *p* = 0.507; Stage II–III: OR: 1.068, 95% CI = 0.754**–**1.512, *p* = 0.711). Sensitivity analyses were performed based on breast cancer molecular subtypes of patients enrolled. With only TNBC or HER2- patients involved, the heterogeneity was dramatically reduced from I-square 88.1% to 0.0%, indicating study by Byrski et al. probably the main source of heterogeneity. And both these two homogeneous subgroups revealed that platinum did not significantly increase pCR rate in either HER2- (OR: 0.935, 95% CI = 0.716**–**1.221, *p* = 0.622) (**Figure 3A**) or TNBC (OR: 1.028, 95% CI = 0.779**–**1.356, *p* = 0.846) (**Figure 3B**) patients.

**Figure 4:**
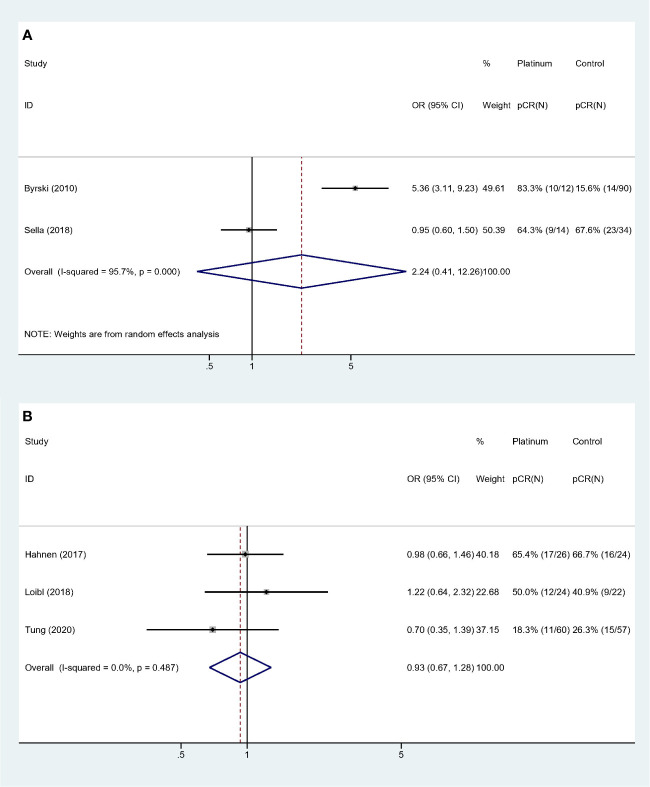
Subgroup analyses according to study design, quality assessment and BRCA mutation status: **(A)** Prospective cohort studies with low quality score and only BRCA1 mutated patients were included. **(B)** Randomized controlled trials with high quality score and both BRCA 1 and 2 mutated patients were included.

Collinearity existed among variable “Study type,” “QS” and “BRCA status”. Forest plots were showed in **Figure 4**. Studies with “PCS design,” “low QS” and “only BRCA1 mutation” suggested platinum did not deliver benefit in neoadjuvant setting (OR: 2.241, 95% CI = 0.410**–**12.259, *p* = 0.352) (**Figure 4A**). For studies with “RCT design,” “high QS,” and “BRCA 1/2 mutation” revealed no significant impact of adding platinum to neoadjuvant therapy (OR: 0.930, 95% CI = 0.674**–**1.283, *p* = 0.659). This subgroup also showed high homogeneity (I-square = 0.0%, *p* = 0.487) (**Figure 4B**).

**Figure 5:**
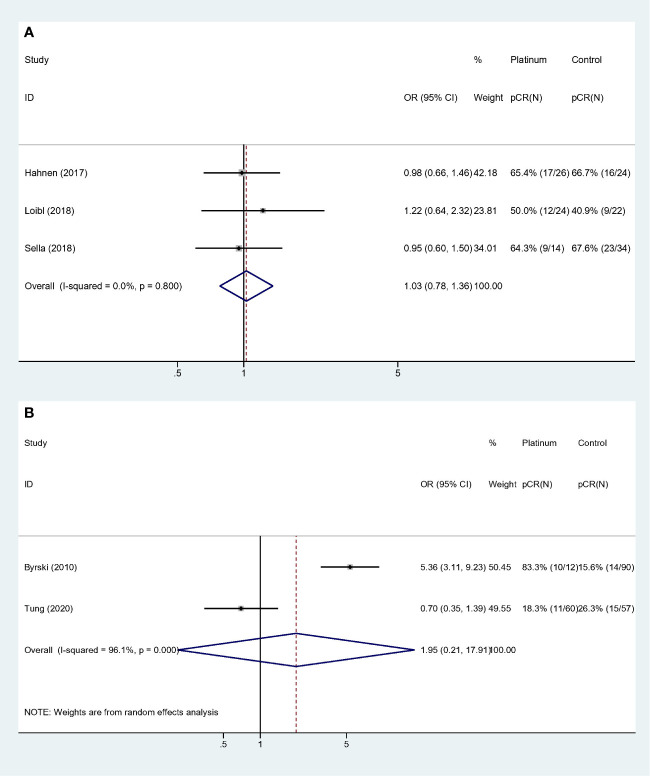
Subgroup analyses according to study population, experiment regimen and platinum usage: **(A)** Studies that included BRCA mutated patients as subgroup and used combination regimen with carboplatin. **(B)** Studies that the whole cohort was BRCA mutated patients and used single agent regimen with cisplatin.

Similarly, collinearity existed among variable “Study population,” “Experiment regimen” and “Platinum used”. Forest plots were showed in **Figure 5**. Studies with “BRCA mutated patients as subgroup,” “combination chemotherapy” and “carboplatin used” showed strong homogeneity (I-square = 0.0%, *p* = 0.800), and the pooled result did not validate the superiority of platinum-based regimen (OR: 1.028, 95% CI = 0.779**–**1.356, *p* = 0.846) (**Figure 5A**). And studies with “all the patients were BRCA1 mutated,” “single-agent chemotherapy,” and “cisplatin used” had the comparable result (OR: 1.950, 95% CI = 0.212**–**17.913, *p* = 0.555) (**Figure 5B**).

### Publication Bias

Potential publication bias was evaluated by Funnel plot with symmetrical appearance (**Supplementary Figure 1**). Begg’s test suggested no significant publication bias (*p* = 0.806).

## Discussion

BRCA mutations account for a large proportion of hereditary breast cancer, and platinum agents and PARP inhibitors are recommended to treat advanced breast cancer in BRCA carriers. However, emerging evidences are not sufficient to support routine addition of platinum to anthracycline- and taxane-based chemotherapy in neoadjuvant setting. The present meta-analysis included five studies with 363 patients and evaluated the efficacy of adding platinum agents in neoadjuvant setting. The pooled results suggested platinum containing regimen did not significantly improve pCR rate (Platinum vs. Control groups: 43.4 vs. 33.9%, OR: 1.340, 95% CI = 0.677**–**2.653, *p* = 0.400). This result was also validated by subgroup analyses of HER2- and TNBC subtypes.

In metastatic setting, TNT trial along with several other studies proved that platinum could incur a higher response rate for metastatic TNBC with BRCA mutation (15–17). The TNT trial enrolled 43 BRCA carriers and demonstrated carboplatin associated with increasing response rate and prolonged progression-free survival for patients with BRCA mutation in first-line metastatic setting (15). Moreover, it was noteworthy that subgroup analysis proved the therapeutic effect was predominantly driven by carboplatin other than taxane. However, in neoadjuvant setting, three RCT trials (GeparSixto, BrighTNESS and INFORM) reached contradictory conclusion (10, 11, 13). The present meta-analysis was concordant with the above three RCT trials and the subgroup analysis of previous meta-analysis that carboplatin did not associated with increased pCR rate for TNBC (OR: 1.028, 95% CI = 0.779**–**1.356, *p* = 0.846) (18). The GeparSixto trial enrolled 291 TNBC and HER2+ breast cancer patients, and evaluated the efficacy and safety profile with the addition of carboplatin to taxane and anthracycline based neoadjuvant regimen. In TNBC subgroup, patients treated with carboplatin revealed significantly higher pCR rate (57%) compared with control group (42.7%, *p* = 0.015). But for BRCA carriers, the pCR rate in both groups were comparable (carboplatin group: 65.4%, control group: 66.7%, OR: 0.94 [0.29-3.05], *p* = 0.920) (13). And in BRCA mutant subgroup of BrighTNess trial, 92 TNBC patients with BRCA mutation was included and platinum based regimen showed the trend with increasing pCR rate (platinum group: 56.5%, control group: 40.9%), however, it did not reach statistical significance (p = 0.344) (10). Unlike the previous two trials that only have subgroup of BRCA mutant patients, INFORM (TBCRC031) trial recruited BRCA mutated patients only and compared cisplatin as single agent with anthracycline and cyclophosphamide (AC) regimen (11). The INFORM trial enrolled 117 patients with BRCA mutation and randomly assigned the participants to receive either cisplatin or AC as neoadjuvant regimen. Cisplatin did not exhibit therapeutic superiority in terms of either pCR or RCB 0/1 rate. The pCR rates were 18% and 26% for cisplatin group and AC group, respectively, indicating no statistically significant improvement with platinum (relative risk [RR]: 0.70, 90% CI = 0.39–1.20). Additionally, the RCB as another endpoint to evaluate the efficacy of neoadjuvant treatment also drew the similar conclusion. The RCB 0 or 1 percentage was also comparable between cisplatin and AC groups [cisplatin (33%) and AC (46%), RR: 0.73, 90% CI = 0.50–1.10]. The INFORM trial had the limitation that the chemotherapy regimens used in this study (single agent cisplatin and AC) were suboptimal for neoadjuvant setting, so the pCR rate in INFORM trial was significantly lower than the other included studies.

In contrast, Study by Bryski et al. suggested cisplatin as single agent neoadjuvant chemotherapy yielded high response rate in BRCA1 mutation carriers (9). It enrolled 102 participants and compared cisplatin as single agent to control group with several doxorubicin and/or taxane based regimens. Cisplatin group had a huge advantage of pCR rate up to 83.3% over control group with only 15.6%. However, the study design was quite different with the other included studies. First, the study recruited patients irrespective of hormone receptor and HER2 status and around 15% of the study population were hormone receptor positive. Second, nearly 1/3 patients had ambiguous or missing HER2 status. Given anti-HER2 agents were regarded as part of the standard treatment of HER2+ breast cancer, missing information on HER2 status could potentially lead to undertreatment and confounded the result. Third, study by Bryski et al. was also considered to be the major source of heterogeneity through sensitivity of the present meta-analysis. Sensitivity analysis revealed strong homogeneity for the rest of the included studies with the exclusion of study by Bryski et al. (**Table 2**, HER2- subgroup, I-square = 0.0%, *p* = 0.496). Additionally, it was noteworthy that this study was the only included study that supported the inclusion of platinum for neoadjuvant chemotherapy. The plausible explanation for the large pCR benefit from platinum could probably be attributed to the fact that this study was published almost a decade before and the regimens largely differed from the other studies. Another reason was a low portion of the participants received doxorubicin in the early phase of the study, which was validated by the earlier report of the same study cohort that pCR rate was only 10% in 41 women with BRCA1 mutation (9, 19). Thus, study by Bryski et al. may probably be an outlier and exclusion of this study may improve the reliability of the overall results. Although it may potentially introduce bias, the conclusion was consistent irrespective of the inclusion of this study. But the other results of subgroup analyses with this study included should be explained with caution.

Theoretically, breast cancer with BRCA mutation was considered to have limited DNA repair function and may be more sensitive to chemotherapeutic agents such as platinum. And this hypothesis was proved in metastatic setting by TNT trial (15). However, the present meta-analysis along with the other three RCT had the contradictory results that platinum did not improve pCR. And this was further proved by survival analysis of Geparsixto trial, the survival benefit of platinum was only observed for patients without a BRCA mutation (13). One plausible explanation for lack of improvement of pCR by platinum in BRCA carriers is that the DNA instability caused by BRCA mutation induces hypersensitivity of tumor response to DNA-damaging agents such as anthracyclines and alkylating agents. And the response was so sensitive that pCR rate easily reached the plateau of the response curve and further increase is difficult to gain by adding new chemotherapeutic agents. This speculation was proved by GeparSixto trial that BRCA carriers had significantly higher pCR rate than non-carriers (BRCA carriers vs. non-carriers, 66.7 vs. 33.4%) (13). And the meta-analysis by Caramelo et al. also showed a similar trend with increased pCR rate of BRCA mutation carriers (58.4%) compared with non-carriers (50.7%) (20). The effect of platinum was probably masked by strong response induced by the other agents. BRCA deficiency may serve as an independent predictor for chemosensitivity, and it should be regarded as a general predictor to chemotherapeutic agents, rather than specific to platinum. The other evidence may come from the POSH study which was by far the largest prospective cohort trial on patients with BRCA mutations (21). It proved the deleterious BRCA mutation increased the breast cancer incidence, but did not compromise overall survival at any timepoint. This could partially attribute to the effective response incurred by standard chemotherapeutic agents.

Our studies had several limitations. First, it based on population data other than individual patient data and restrained our ability to conduct subgroup analyses on several critical clinicopathological variables, such as different intrinsic subtypes and chemotherapy regimens. Second, although three RCT trials were included, two of the three studies only provided subgroup with BRCA mutation. Subgroup without prespecified design may probably have unbalanced baseline characteristics and insufficient sample size, thus, the statistical power may be not strong enough to support the conclusion. Third, due to limited studies included, meta regression was unable to perform. Finally, survival data were missing in most of included studies. It was of note that increasing pCR rate would not necessarily transfer to survival benefit.

Future large-scale randomized control trial would be the optimal choice to further validate the above conclusion. And given the low incidence of BRCA mutation and time-consuming recruitment process of BRCA related clinical trials, real-world studies with large sample size and multi-center involved would be a reasonable alternative.

## Conclusion

Our meta-analysis suggested that the addition of platinum to neoadjuvant chemotherapy did not significantly improve pCR rate for patients with BRCA mutations. Further analyses with individual patient data and survival data may provide more robust evidence on therapeutic value of platinum in breast cancer and facilitate personalized medicine.

## Data Availability Statement

All datasets presented in this study are included in the article/**Supplementary Material**.

## Author Contributions

C-JW, YX, QS and C-GL designed the project. C-JW, YX, YL, QS, and C-GL performed the literature search and data acquisition. C-JW, YX, H-JZ, Y-DZ, and YZ performed data extraction and statistical analyses. X-HZ, FM, and XH performed the statistical analyses for heterogeneity investigation. C-JW, YX, and YL supported the writing of the paper. All authors contributed to the article and approved the submitted version.

## Funding

This study was funded by Key Projects in the National Science and Technology Pillar Program during the Twelfth Five-year Plan Period (no. 2014BAI08B00), Beijing Municipal Science and Technology Project (no. D161100000816005), and State Key Laboratory of Medicinal Chemical Biology (NanKai University) (no. 2019014). The funding agencies had no role in the design or conduct of the study.

## Conflict of Interest

The authors declare that the research was conducted in the absence of any commercial or financial relationships that could be construed as a potential conflict of interest.

## Supplementary Material

Supplementary Figure 1 Funnel plot for potential publication bias.
