# Supplementary figures and images for: Platinum-Based Neoadjuvant Chemotherapy for Breast Cancer With BRCA Mutations: A Meta-Analysis
Source: Front Oncol. 2020 Nov 9;10:592998. doi: 10.3389/fonc.2020.592998 (PMC7693629; doi:10.3389/fonc.2020.592998)

Begg's funnel plot with pseudo 95% confidence limits

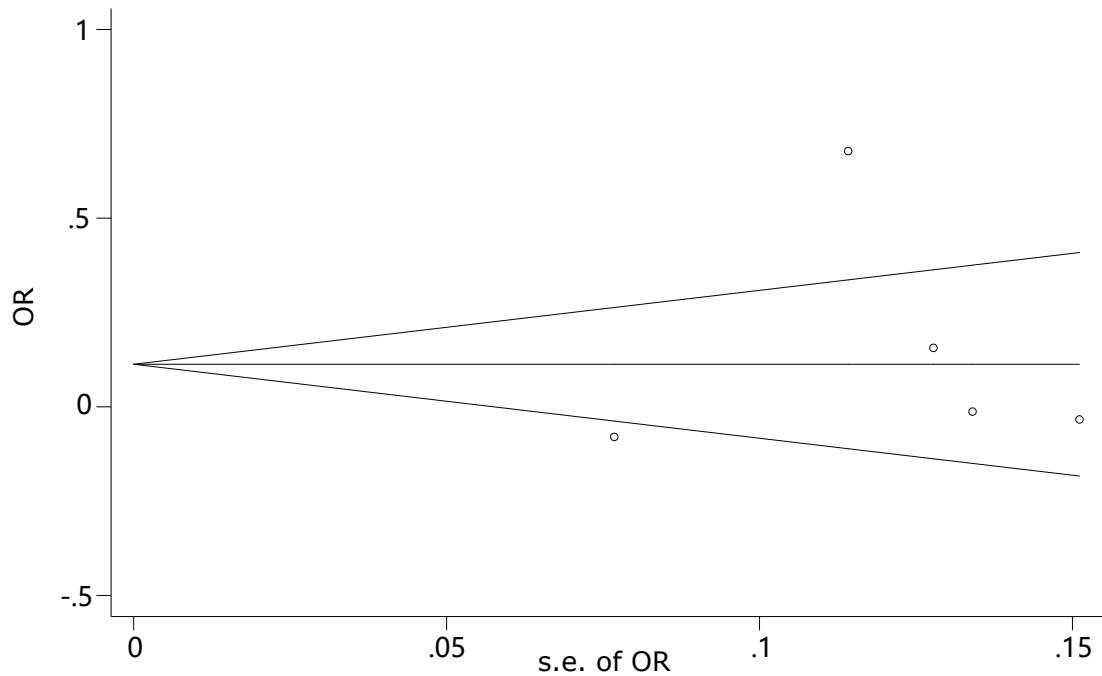

Supplement: Supplementary Figure 1 — Funnel plot for potential publication bias. [file DataSheet_1.pdf]
