# Supplementary material for: Platinum-Based Neoadjuvant Chemotherapy for Breast Cancer With BRCA Mutations: A Meta-Analysis
Source: Front Oncol. 2020 Nov 9;10:592998. doi: 10.3389/fonc.2020.592998 (PMC7693629; doi:10.3389/fonc.2020.592998)
Supplement: Supplementary file 2 [file Table_1.doc]

Sup. Table 1 Quality assessment of included studies

| Study | QS^*^ | | | Quality  Assessment^&^ |
| --- | --- | --- | --- | --- |
|  | Reviewer 1 | Reviewer 2 | Final QS^#^ |  |
| **Byrski 2010** | 92.73 | 91.82 | 92.28 | Low |
| **Hahnen 2017**  **(GeparSixto)** | 98.18 | 97.27 | 97.73 | High |
| **Loible 2018**  **(BrighTNess)** | 97.27 | 96.36 | 96.82 | High |
| **Sella 2018** | 89.09 | 87.27 | 88.18 | Low |
| **Tung 2020 (INFORM)** | 99.09 | 97.27 | 98.18 | High |

^*^ Quality score (QS) was generated according to the items in STROBE Checklist. Each item in the STROBE Checklist was scored using an ordinal scale (1-5, with 1=Worst, 5=Best) by two independent reviewers (C.J. Wang and Y. Lin).

^#^ The final QS were the average of scores generated by each reviewer and expressed as percentages, ranging 0–100%.

^&^ The cutoff value to divide included studies into low-quality and high-quality groups was set as the mean of the QS of all the included studies.
